# Transactions of the New Jersey State Medical Society for 1859

**Published:** 1860-11

**Authors:** 


ART. XIV.—*Transactions of the New Jersey State Medical Society for* 1859. pp. 95. Newark, New Jersey.

IT is an interesting fact, and one which is not generally known, that the Medical Society of New Jersey is the oldest State medical institution of the kind in America, having been founded in 1766. The first meeting of the Society was held at New Brunswick in July of that year, and was attended by fourteen physicians, all animated, to use the language of Dr. Coleman, its late President, "by a laudable desire to elevate the low state of medicine in New Jersey, and to advance its dignity." The address of this gentleman, delivered at the meeting at Trenton, is characterized by good taste, and strongly urges upon the members of the Society the importance of steady professional progress.

The Transactions are, in the main, meagre and unimportant, as is, indeed, too generally the case with the transactions of similar bodies, especially in this country. Any progress, however, is better than complete inertia; and we feel confident that the effect of Dr. Coleman's able address will not be lost upon his associates, but that it will serve to rouse them to increased exertion in behalf of the great cause in which they are engaged. From some hints in the Transactions, we infer that the profession of the State is not perhaps quite as harmonious, as a body, as it ought to be. This is to be regretted, because not only the profession but also the public will be sufferers by it. We shall be glad, from time to time, to transfer some of the Society's matter to the pages of the REVIEW.

